# Characterization of corneal stromal stem cells with the potential for epithelial transdifferentiation

**DOI:** 10.1186/scrt226

**Published:** 2013-06-24

**Authors:** Khurram Hashmani, Matthew James Branch, Laura Elizabeth Sidney, Permesh Singh Dhillon, Megha Verma, Owen Douglas McIntosh, Andrew Hopkinson, Harminder Singh Dua

**Affiliations:** 1 The Division of Ophthalmology and Visual Sciences, Queen’s Medical Centre Campus, University of Nottingham, Nottingham, UK

**Keywords:** Corneal stroma, Corneal epithelium, Mesenchymal stromal cells, Cell transdifferentiation, CD34, Epithelial-mesenchymal transition

## Abstract

**Introduction:**

The corneal stroma is being increasingly recognized as a repository for stem cells. Like the limbal and endothelial niches, stromal stem cells often reside in the peripheral cornea and limbus. These peripheral and limbal corneal stromal cells (PLCSCs) are known to produce mesenchymal stem cells *in vitro.* Recently, a common corneal stromal and epithelial progenitor was hinted at. This study aims to examine the stem cell potential of corneal stromal cells and to investigate their epithelial transdifferentiation ability.

**Methods:**

PLCSCs were grown in traditional Dulbecco modified Eagle medium (DMEM)-based keratocyte culture medium and an M199-based medium and analyzed for a profile of cell-surface markers by using flow cytometry and differentiated into mesenchymal phenotypes analyzed with quantitative polymerase chain reaction (qPCR) and histologic staining. PLCSCs in M199 were subsequently divided into subpopulations based on CD34 and CD105 expression by using fluorescence- activated cell sorting (FACS). Subpopulations were characterized by marker profile and mesenchymal differentiation ability. Both whole PLCSCs and subpopulations were also cultured for epithelial transdifferentiation.

**Results:**

Cells cultured in M199 demonstrated a more stem-like cell-surface marker profile, and the keratocyte marker CD34 was retained for several passages but absent in cells cultured in DMEM. Cells cultured in M199 also exhibited a greater mesenchymal differentiation potential, compared with DMEM. PLCSCs could be divided into CD34^+^CD105^+^, CD34^-^CD105^+^, and CD34^-^CD105^-^ subpopulations, of which CD34^+^CD105^+^ cells were the most stemlike with regard to marker expression and mesenchymal differentiation potential. Subpopulations of PLCSCs exhibited differing abilities to transdifferentiate into epithelial phenotypes. Cells that were initially CD34^+^CD105^+^ showed the greatest differentiation potential, producing CK3^+^ and CK19^+^ cells, and expressed a range of both epithelial progenitor (*HES1, FRZB1, DCT, SOD2, ABCG2, CDH1, KRT19*) and terminally differentiated (*DSG3, KRT3, KRT12, KRT24*) genes.

**Conclusions:**

Culture medium has a significant effect on the phenotype and differentiation capacity of PLCSCs. The stroma contains a heterogeneous cell population in which we have identified CD34^+^ cells as a stem cell population with a capacity for mesenchymal and epithelial differentiation.

## Introduction

Corneal blindness is a leading worldwide cause of treatable vision loss [1]. Trauma to the cornea can occur from a wide range of environmental factors, including chemical and thermal burns, mechanical and surgical trauma, or microbial infection [2,3]. Specialized cellular and structural organization is essential for the corneal transparency required for effective vision [4-7]; the cornea poses unique therapeutic challenges. Donor shortages and problems with immune rejection have propelled the development of regenerative medicine strategies for the cornea. Current and potential treatments are diverse; approaches include partial or total corneal replacements, the use of cellular or acellular constructs, and synthetic or biologic materials [8-12].

The corneal stem cell niche is located at the limbus, the boundary of the cornea, and contains both corneal epithelial [13,14] and stromal stem cells [15-18]. However, the term limbal stem cells (LSCs) is applied exclusively to the corneal epithelial progenitors. LSC transplantation is used routinely to treat epithelial deficiencies in the cornea [3,9,19]. This can involve direct transplantation of limbal tissue for *in situ* epithelial regeneration, or indirect transplantation of *ex vivo* expanded sheets of replacement cells [3,9,20]. Both treatment strategies can comprise autologous [4,9,20] or allogeneic [19] material.

Corneal and limbal epithelium is supported by a mesenchymal stroma [21,22], which contains cells conventionally known as keratocytes. Keratocytes normally remain quiescent [23,24] and exhibit a dendritic morphology with extensive cellular contacts [25,26]. These cells maintain corneal stromal transparency at a structural level by producing collagen lamellae and proteoglycans, including keratocan, decorin, lumican, and mimecan [23,27-34]. At the intracellular level, transparency is aided by the production of crystallins, aldehyde dehydrogenase class 1 (ALDH1) and transketolase [35-37]. These characteristic proteins can be used to identify keratocytes, along with cell-surface markers CD133 and CD34 [24,38,39].

The perception of keratocytes and their role within the corneal and limbal stroma is shifting as other properties are being attributed to them. Recently, we demonstrated that cultured stromal cells of the limbus and peripheral cornea (PLCSCs)*,* produce a mesenchymal stem cell (MSC) population [15], as described by the International Society for Cellular Therapy (ISCT) [40]. Subsequent research on these MSCs has shown that they may provide a supportive niche for epithelial stem cells [41], similar to the role of MSCs in bone marrow [42,43], and that they possess the immunosuppressive properties demonstrated by MSCs from other sources [44]. Bray *et al.*[45] found the typical keratocyte culture conditions of Dulbecco modified Eagle medium (DMEM), supplemented with 10% fetal bovine serum (FBS), produced suboptimal culture conditions for MSCs, and that other media types may generate a more stemlike phenotype. What remains uncertain is whether the cell-culture medium produces a change in cellular phenotype, or if specific subpopulations present in PLCSCs are favored in different media.

Although the use of corneal stromal stem cells in corneal regeneration is less established than that of LSCs, increasing evidence suggests an effectiveness in this area, particularly in innovative tissue-engineering strategies [46-48]. PLCSCs may therefore play a direct role in the provision of cells for corneal maintenance and regeneration. Within PLCSCs, CD34^+^ keratocytes are of particular interest, as increasing evidence suggests an association between the expression of CD34 and a common mesenchymal/epithelial progenitor in other tissues [49-51]. MSC-epithelial transdifferentiation also has been reported [52-55], and CD34 is commonly used as a marker of skin epithelial progenitors that reside in the stroma of the dermis [56-58]. Therefore, mesenchyme-derived limbal stromal progenitors may play a role in corneal epithelial regeneration. In support of this, we recently published evidence that suggests that CD34^+^ keratocytes spontaneously co-express the corneal epithelial marker cytokeratin (CK) 3 [59].

Herein, we compare the stem cell phenotype of PLCSCs in our previously published culture conditions [15] with those cultured in the traditional keratocyte medium and show that despite its widespread use and lower serum content, the keratocyte medium is inferior at maintaining an MSC phenotype. We also demonstrate the heterogeneity of PLCSCs by isolating distinct subpopulations, including CD34^+^ keratocytes. We then establish that the CD34^+^ subpopulation shows enhanced epithelial transdifferentiation in comparison to the other subpopulations.

## Materials and methods

### Isolation of peripheral and limbal corneal stromal cells

Use of human donor tissue for research was approved by the local ethics research committee (NRES Committee East Midlands-Nottingham 1, 07/H0403/140). and in accordance with the tenets of the declaration of Helsinki, after consent obtained from the donors or their relatives.

PLCSCs were isolated from corneal rims, as previously described [15,60]. In brief, the epithelial and endothelial layers were removed by mechanical scraping, and the remainder of the limbus/tissue was cut into small pieces and digested in 0.1 mg/ml collagenase type IA (Sigma Aldrich, Dorset, Gillingham, UK). The tissue was incubated for approximately 18 hours at 37.0°C, 5% CO_2_, 95% humidity, and filtered with a 41-μm nylon net filter (Fisher Scientific, Leicestershire, UK) to remove debris. Culture medium was added to the collagenase filtrate solution before centrifugation at 450 *g* for 6 minutes. The supernatant was decanted, and the cell pellet resuspended in the appropriate culture medium.

### Cell culture

PLCSCs were cultured in one of two culture media. First was a standard keratocyte medium (KM), [17,46] consisting of DMEM (Gibco, Invitrogen, Paisley, UK), supplemented with 10% vol/vol heat-inactivated FBS (Fisher Scientific), 0.02 μg/ml gentamicin, 0.5 ng/ml amphotericin B (Gibco), 4.5 μg/ml insulin, human recombinant (Gibco), and 0.5% vol/vol DMSO (Sigma Aldrich). The second was a medium previously shown to support the expansion of MSCs [15,61-63] (MM), consisting of M199 medium (Sigma) supplemented with 20% vol/vol heat-inactivated FBS, 2.5 μg/ml antibiotic solution, Plasmocin (Autogen Bioclear, Wiltshire, UK), 0.02 μg/ml gentamicin, 0.5 ng/ml amphotericin B (Gibco), and 1.59 m*M* L-glutamine (Sigma Aldrich).

All extracted PLCSCs were initially cultured in 25 cm^2^ culture flasks (Fisher Scientific); this was considered to be passage 0 (P0), and the medium was changed every 2 days. Cells were passaged at 80% confluence at a 1:3 ratio, as previously described [15].

### Sample preparation for flow-cytometry analysis and cell sorting

PLCSCs and subpopulations were prepared, analyzed, and sorted by using protocols previously described [15]. In brief, cells for analysis were suspended in phosphate-buffered saline (PBS) and fixed by using 3% vol/vol formaldehyde (Sigma Aldrich) for 5 minutes and subsequently washed. PLCSCs for sorting were suspended in a minimal volume of corresponding culture medium. Cells were then incubated with the appropriate primary conjugated antibodies for 30 minutes, washed, and resuspended. PLCSCs were analyzed by using the Epics Altra Flow Cytometer (Beckman Coulter, London, UK). Antibodies were as follows: CD11b, CD13, CD19, CD29, CD34, CD44, CD45, CD49b, CD49d, CD49e, CD105, HLA-ABC, and HLA-DR (Beckman Coulter), CD49f, CD104, CD106, and cytokeratin 14 (AbD Serotec, Oxford, UK), CD73 (R&D Systems, Foster City, CA, USA), CD90 (BD Pharmingen, Oxford, UK), CD133/2, and CD271 (Miltenyi Biotec, Surrey, UK), ABCG2 (Santa Cruz, Middlesex, UK), Stro-1 (Biolegend, Cambridge, UK), Keratin 3/76 (CK3; Millipore, Fisher Scientific), Cytokeratin 14 (CK14, AbD Serotec), Cytokeratin 19 (CK19), vimentin (Abcam, UK), and ABCG2 (Santa Cruz, UK).

PLCSCs were sorted between P0 and P1 by using FACS with a MoFlo XDP Cell Sorter (Beckman Coulter). After FACS, subpopulations were cultured for a further three passages for cell-surface marker (CSM) analysis and differentiation. Postanalysis data were plotted by using WEASEL version 3.0, as previously described [15]. Isotype controls were used as negative controls, and the threshold set to 0.5% for the percentage of positive cells.

### Differentiation and histologic staining

PLCSCs were seeded at a density of 1.04 × 10^4^ cells/cm^2^ in six-well plates at P3 and differentiated into adipogenic, chondrogenic, or osteogenic differentiation medium, for 21 days, as previously described [15,64]. Cells were stained with Oil Red O (Sigma Aldrich), Alcian blue (HD Supplies, UK), and Alizarin red (Sigma Aldrich) for adipogenic, chondrogenic, and osteogenic differentiation, respectively. Cells were visualized under an inverted light microscope (Nikon Eclipse TS100 Light Microscope, Japan), and images captured with a digital camera (Nikon D70s).

### Epithelial transdifferentiation

FACS-isolated subpopulations were seeded at a density of 1.04 × 10^4^ cells/cm^2^ and cultured in MM. At 90% confluence, MM was switched to CnT-20 medium (CellnTec Advanced Cell Systems, Switzerland). Negative controls were maintained in MM and cultured alongside. Culture medium was changed every 2 days, and cells were cultured for 14 days. Cells were imaged under a light microscope, as previously described [15].

### RNA extraction, cDNA synthesis, and quantitative PCR

RNA was isolated from cells by using the RNeasy Mini Kit (Qiagen, UK), and each sample was analyzed in triplicate in accordance with the manufacturer’s protocol: 1 ng/μl of extracted RNA was used for cDNA synthesis by using the QuantiTect Reverse Transcription Kit (Qiagen, UK) according to the manufacturer’s protocol.

Inventoried Taqman assays (Applied Biosystems, Warrington, UK) were used for the genes as follows: adipogenesis, fatty acid synthase (*FASN*), perilipin (*PLIN*) and peroxisome proliferator-activated receptor gamma (*PPARG*); chondrogenesis, cartilage oligomeric matrix protein (*COMP*), aggrecan (*ACAN*), and Sry-related HMG box 9 (*SOX-9*); and osteogenesis, bone morphogenetic protein 4 (*BMP4*), bone morphogenetic protein 6 (*BMP6*), and osteoclastogenesis inhibitory factor (*OPG*). Corneal epithelial progenitor genes; hairy and enhancer of split 1 (*Drosophila*), (*HES1*), frizzled-related protein (*FRZB1*), dopachrometautomerase (dopachrome delta-isomerase, tyrosine-related protein 2) (*DCT*), superoxide dismutase 2, mitochondrial (*SOD2*), ATP-binding cassette, subfamily G member 2 (*ABCG2*), E-cadherin (cadherin 1 type 1, *CDH1*), and keratin 19 (*KRT19*). Differentiated corneal epithelial genes; keratin 3 (*KRT3*), keratin 12 (*KRT12*), keratin 24 (*KRT24*), desmoglein 3 (*DSG3*). Housekeeping gene; *18S rRNA*.

Amplification was performed by using the Mx2005P multicolor 96-well PCR system (Stratagene, Agilent Technologies, Cheshire, UK) with parameters of 95°C for 10 minutes) followed by 40 cycles of 95°C for 30 seconds, 55°C for 1 minute, and 72°C for 30 seconds. Data analysis was performed by using MxPro software, version 4.2 (Stratagene, UK) to measure the threshold cycle (C_t_) for each reaction. The mean C_t_ value was established by using triplicate C_t_ values, and analysis was completed by using the ΔΔC_t_ method [65].

## Results

### Cell-surface marker expression

**Figure 1:**
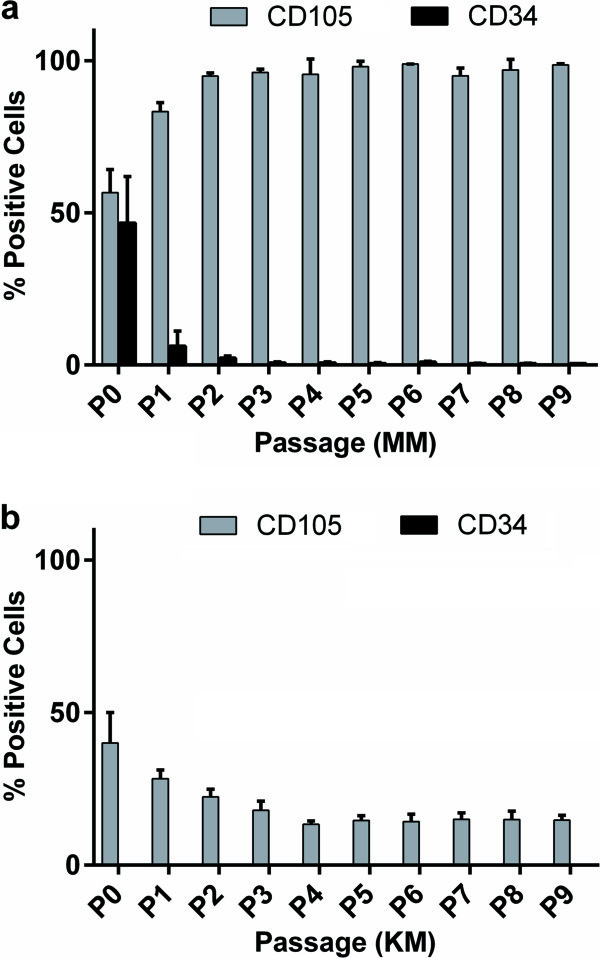
**Effect of culture medium on percentage of PLCSCs expressing CD105 and CD34.** PLCSCs were cultured in either KM or MM, for up to nine passages. At each passage, cells were analyzed with flow cytometry for the presence of CD34 and CD105. Values are represented as mean ± SD; flow cytometry was performed on three separate donor-cell populations.

Initially, the PLCSCs were cultured in either KM or MM, and the expression of CD105 and CD34 were analyzed by using flow cytometry at each passage, up to passage 9 (Figure 1). In MM, 55% of cells expressed CD105 at P0, which increased to >95% at P3, remaining stable up to P9 (Figure 1a). However, in KM, the numbers of PLCSCs expressing CD105 at P0 were proportionally lower (40%), and decreased to 18% at P3 (Figure 1b); between P4 and P9, CD105 was expressed in an average 15% of PLCSCs cultured in KM (Figure 1b). CD34 expression was seen only in PLCSCs cultured in MM (Figure 1a). Expression at P0 averaged 47%, but decreased rapidly during culture and subsequent passaging.

This apparent heterogeneity within PLCSCs in different media was further assessed by using a panel of cell-surface markers (CSMs). Markers included the ISCT criteria for MSC, additional markers chosen based on their inclusion as MSC markers in the literature, and control markers for ocular surface epithelium (Table 1). Analysis of PLCSCs cultured in MM conformed to the ISCT CSM criteria for MSCs. At P3, ≥95% of cells were positive for CD73, CD90, and CD105 and were negative (≤2%) for CD11b, CD19, CD34, CD45, and HLA-DR. In contrast, PLCSCs cultured in KM did not conform to the ISCT criteria; The cells did not meet the positive threshold values (≥95%) for CD73 and CD105, with CD105 expressed only in an average 21% of PLCSCs, compared with 97% for cells cultured in MM. Further differences were found in marker expression between cells cultured in the different media; PLCSCs cultured in KM showed reduced proportions of ABCG2, CD13, CD49e, HLA-ABC, and vimentin compared with cells grown in MM. PLCSCs cultured in KM and MM did not display any detectable levels of CK3, CK14, or CK19, indicating no epithelial contamination.

**Table 1:** Cell-surface marker profiling of PLCSCs and isolated subpopulations for ISCT criteria and additional markers

**Cell-surface markers**	**Mean % of positive cells cultured in**		**Mean % of positive cells in subpopulation**		
	**MM**	**KM**	**A**	**B**	**C**
**ISCT Ppsitive criteria (≥95%)**_**1**_					
CD73	**97.13** ± 0.81 **✓**	**82.47** ± 2.73 **✕**	**97.13** ± 0.81 **✓**	**64.00** ± 4.00 **✕**	**73.00** ± 3.00 **✕**
CD90	**98.13** ± 2.71 **✓**	**99.97** ± 0.06 **✓**	**99.47** ± 0.40 **✓**	**99.97** ± 0.06 **✓**	**97.67** ± 1.53 **✓**
CD105	**96.67** ± 1.53 **✓**	**21.33** ± 2.08 **✕**	**72.00** ± 6.00 **✕**	**50.00** ± 10.00 **✕**	**46.67** ± 20.82 **✕**
ISCT negative criteria (<2%)					
CD11b	**- ✓**	**- ✓**	**- ✓**	**- ✓**	**- ✓**
CD19	**- ✓**	**0.54** ± 0.42 **✓**	**- ✓**	**0.54** ± 0.42 **✓**	**- ✓**
CD34	**- ✓**	**- ✓**	**- ✓**	**- ✓**	**- ✓**
CD45	**- ✓**	**1.80** ±0.26 **✓**	**4.00** ± 2.00 **✕**	**- ✓**	**- ✓**
HLA DP,DQ,DR	**- ✓**	**- ✓**	**- ✓**	**- ✓**	**- ✓**
Additional Markers					
ABCG2	**2.05** ± 1.69	**0.53** ± 0.04	**10.00** ± 2.00	**-**	**-**
CD13	**56.67** ± 28.87	**43.00** ± 1.00	**70.00** ± 17.32	**2.20** ± 0.20	**3.27** ± 0.25
CD29	**97.93** ± 2.55	**97.23** ± 2.66	**97.93** ± 2.55	**96.90** ± 2.15	**96.23** ± 1.33
CD44	**97.00** ± 1.00	**90.33** ± 0.58	**96.00** ± 2.00	**99.33** ± 0.58	**99.00** ± 1.00
CD49e	**77.00** ± 1.00	**50.00** ± 5.00	**59.07** ± 12.18	**48.33** ± 1.53	**30.00** ± 10.00
CD49f	**-**	**-**	**-**	**-**	**-**
CD104	**-**	**-**	**-**	**-**	**-**
CD106	**2.60** ± 0.26	**0.6** ± 0.26	**-**	**1.50** ± 0.46	**1.30** ± 0.03
CD133	**0.88** ± 0.15	**0.59** ±0.09	**0.88** ± 0.15	**0.59** ± 0.09	**0.66** ± 0.15
CD271	**-**	**0.56** ±0.48	**4.80** ± 1.85	**-**	**-**
HLA ABC	**97.90** ± 1.14	**84.00** ± 1.00	**42.00** ± 3.00	**7.00** ± 2.00	**4.00** ± 1.00
Stro-1	**0.56** ± 0.27	**0.47** ± 0.41	**0.56** ± 0.27	**-**	**-**
Vimentin	**17.67** ± 2.52	**8.00** ±1.00	**13.33** ± 5.77	**14.00** ± 2.00	**10.00** ± 2.00
Control markers					
CK 3/12	**0.54** ± 0.23	**-**	**0.60** ± 0.23	**-**	**-**
CK 14	**0.63** ± 0.21	**-**	**0.81** ± 0.21	**-**	**-**
CK 19	**0.78** ± 0.29	**-**	**0.72** ± 0.29	**-**	**-**

PLCSCs cultured in MM and KM were analyzed for cell-surface markers at P3. Subpopulations (A: CD34^+^CD105^+^, B: CD34^-^CD105^+^, C: CD34^-^CD105^-^) were analyzed after cell sorting and culture in MM up to P3. **✓**denotes that the cells have fulfilled the ISCT criteria, **✓**denotes that the cells did not conform to the ISCT criteria. Values of ≤0.5% are denoted by dash (-). Values are represented by mean standard deviation (in brackets). Flow cytometry was performed on three separate donor-cell populations.

To investigate the heterogeneity of PLCSCs further, cells were cultured in MM and sorted on the basis of their respective CD34 and CD105 positivity. Three subpopulations were isolated: CD34^+^CD105^+^, referred to as subpopulation A; CD34^-^CD105^+^ (subpopulation B); and CD34^-^CD105^-^ (subpopulation C). Subpopulations cultured in MM for a further three passages after isolation were analyzed for the panel of CSM previously described (Table 1). Individually, isolated cell subpopulations did not conform to the ISCT CSM criteria. Although initially FACS isolated for CD34, after subsequent culture, subpopulation A no longer contained CD34^+^ cells, and the percentage of CD105^+^ cells had decreased to about 70%, falling below the ISCT positive threshold. Subpopulation A contained greater numbers of cells expressing HLA ABC, CD13, and CD49e, when compared with subpopulations B and C, and were the only subpopulation to possess ABCG2-, CD45-, and CD271-positive cells. Subpopulation B remained CD34^-^, and CD105 positivity had decreased to 50%. Subpopulation C also remained CD34^-^ after culture but had regained CD105 expression in an average of 47% of cells. Subpopulations A, B, and C did not display any detectable levels of CK3, CK14, or CK19, indicating no epithelial contamination.

### Mesenchymal lineage differentiation potential

**Figure 2:**
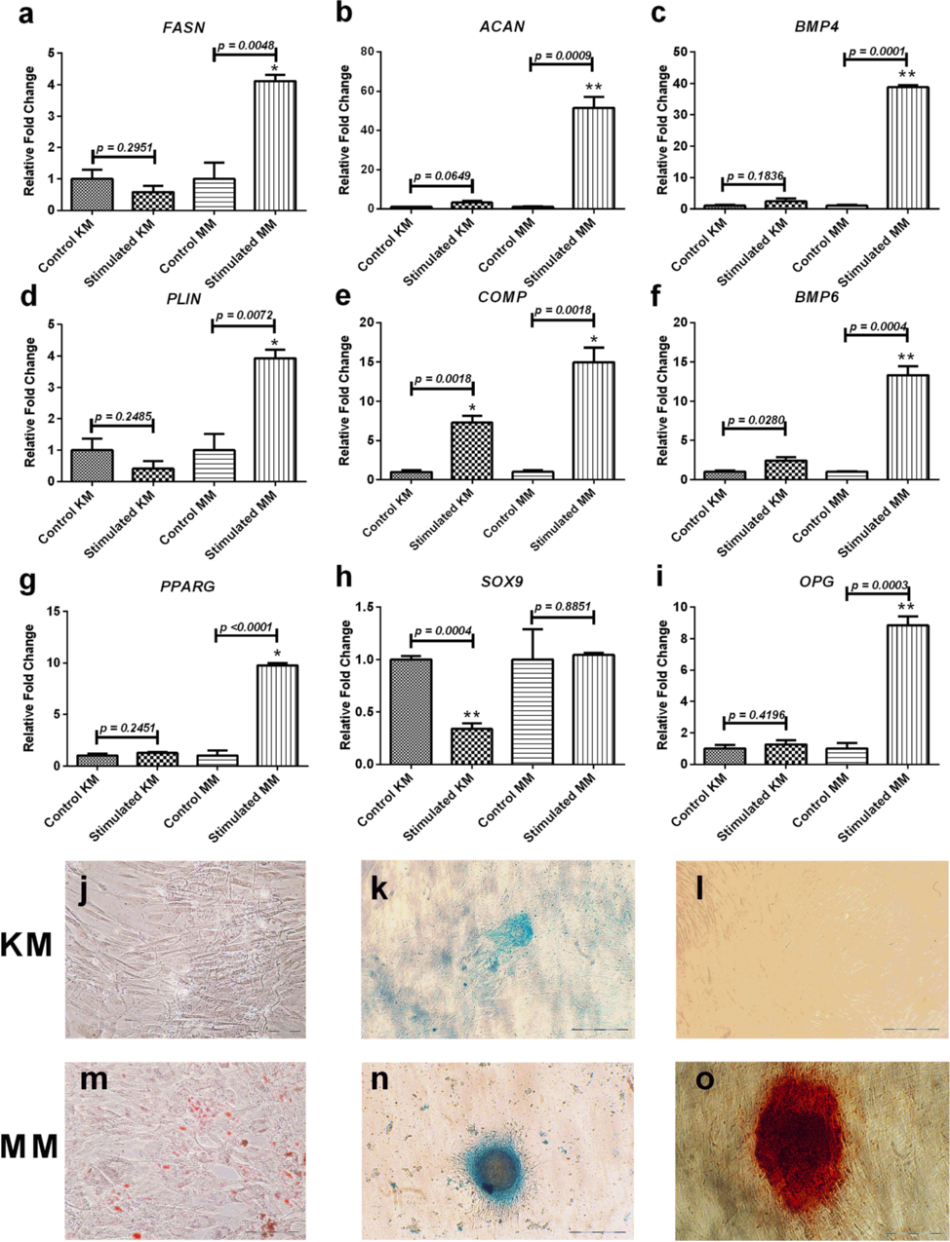
**Effect of culture medium on the mesenchymal differentiation potential of PLCSCs.** PLCSCs were cultured in KM or MM and put through adipogenic, chondrogenic, and osteogenic differentiation protocols, and comparative qPCR analysis (**a** through **i**) and histologic staining (**j** through **o**) were performed. Gene expression and staining for adipogenesis (a, *FASN*, d, *PLIN;*, g, *PPARG,* j and m, Oil Red O staining); chondrogenesis (b, *ACAN*, e, *COMP*, h, *SOX9*, k and n, Alcian blue staining); osteogenesis (c, *BMP4*, f, *BMP6*, I, *OPG*, l and o, Alizarin red staining). Values represented as mean ± SD, experiment repeated in triplicate, each with *n* = 3. *P* values for gene expression are indicated, and statistically significant values are represented (*P < 0.01; **P < 0.001). Experiment was repeated in triplicate. Positive adipogenic, chondrogenic, and osteogenic staining are represented by the presence of lipids (red), glycosaminoglycans (blue), and extracellular calcium deposits (red), respectively. Magnification, 400×.

The ability of PLCSCs to differentiate into adipogenic, osteogenic, and chondrogenic lineages was assessed in both KM and MM (Figure 2). PLCSCs differentiated into all three lineages when cultured in MM; however, they demonstrated negligible differentiation potential when cultured in KM. In adipogenic MM cultures, Oil Red O-stained fat vacuoles were observed throughout but were not present in stimulated KM and negative controls. Adipogenic genes were significantly upregulated when stimulated in MM cultures, with increases in the relative transcription of PPARG (10-fold change (fc)), PLIN (4-fc), and FASN (4-fc), when compared with unstimulated controls. No upregulation of adipogenic genes was seen in cells differentiated in KM cultures. Chondrogenesis was observed with Alcian blue staining of glycosaminoglycans. Deep staining of chondrogenic nodules can be seen in cultures cultured in MM, whereas only light staining was seen in KM cultures. Figure 2k and 2n show high-magnification images of individual chondrogenic nodules; MM cultures showed nodules, such as these, across the plate, but in KM, few nodules were seen. Chondrogenic genes were also seen to be upregulated in stimulated MM, with significant levels of *ACAN* (50-fc) and *COMP* (15-fc) compared with KM. The *SOX9* gene showed no significant upregulation in any medium. *COMP* was the only gene upregulated in chondrogenic KM cultures (7-fc). Osteogenic differentiation was shown by Alizarin red staining of calcium deposition in stimulated MM but not in KM. Figure 2l and 2o show high-magnification images of an individual bone nodule in MM, but no staining was seen in KM. Bone nodules were numerous across the MM cultures. Significant upregulation of BMP4 (40-fc), BMP6 (13-fc), and OPG (9-fc) also occurred in stimulated MM, compared with controls. Stimulated KM showed only small upregulation of BMP6 compared with controls.

**Figure 3:**
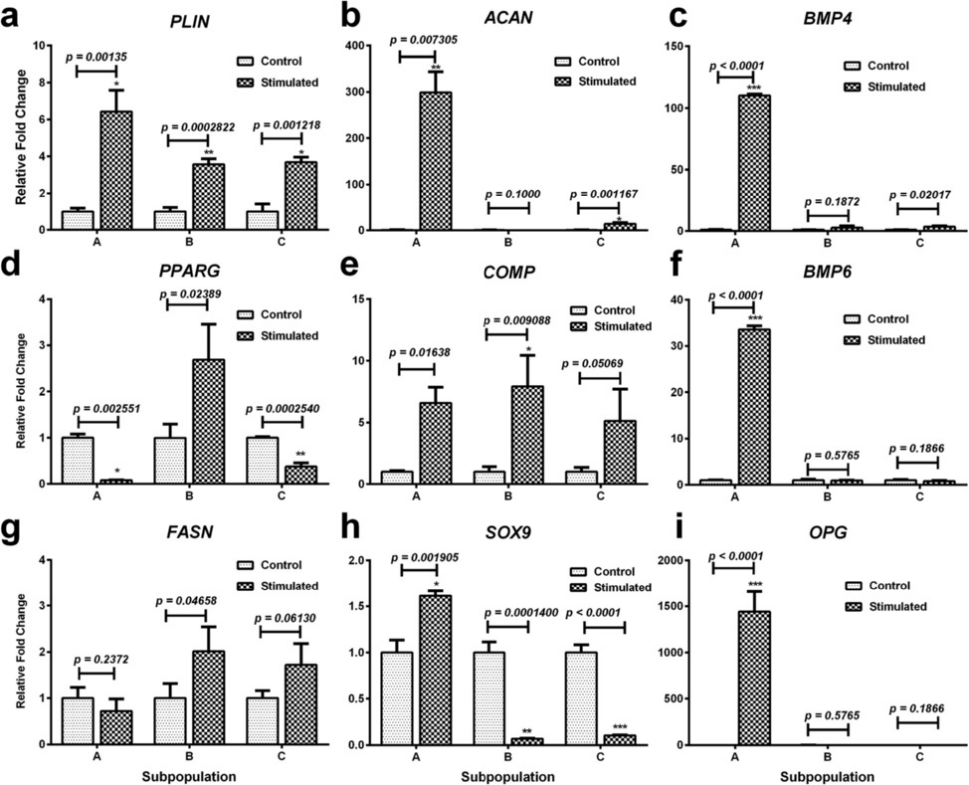
**Mesenchymal differentiation potential of subpopulations of PLCSCs.** PLCSCs were sorted into subpopulations (A, CD34^+^CD105^+^; B, CD34^-^CD105^+^; C, CD34^-^CD105^-^) and put through adipogenic, chondrogenic, and osteogenic differentiation protocols and comparative qPCR analysis (**a** through **i**) were performed. Gene expression for adipogenesis (a, *FASN*; d, *PLIN*; g *PPARG*; chondrogenesis (b, *ACAN*; e, *COMP*; h, *SOX9*); osteogenesis (c, *BMP4*; f, *BMP6*; I, *OPG)*. Values are represented as mean ± SD, and experiments were repeated in triplicate, each with *n* = 3. *P* values for gene expression are indicated, and statistically significant values are represented (*P < 0.01; **P < 0.001).

The mesenchymal differentiation potential of the different subpopulations also was assessed (Figure 3). Subpopulation A demonstrated significant upregulation of the *PLIN* (6-fc) gene but not *PPARG* and *FASN*. Subpopulation B showed general upregulation of all adipogenic genes, although only PLIN (4-fc) was statistically significant. PLIN (4-fc) was also significantly upregulated in subpopulation C, whereas PPARG was downregulated, and FASN was not significant with respect to the unstimulated control. Upregulation of the chondrogenic genes *ACAN* (300-fc), *COMP* (7-fc), and *SOX9* (1.5-fc) was seen in subpopulation A, whereas only *COMP* (8-fc) was significantly upregulated in subpopulation B. *ACAN* (14-fc) and *COMP* (5-fc) were both upregulated in subpopulation C. Subpopulation A demonstrated significant upregulation of the osteogenic genes *BMP4* (110-fc), *BMP6* (34-fc), and *OPG* (1441-fc) compared with unstimulated control. Both subpopulations B and C showed little significant change in osteogenic genes.

### Mesenchymal-epithelial transdifferentiation

**Figure 4:**
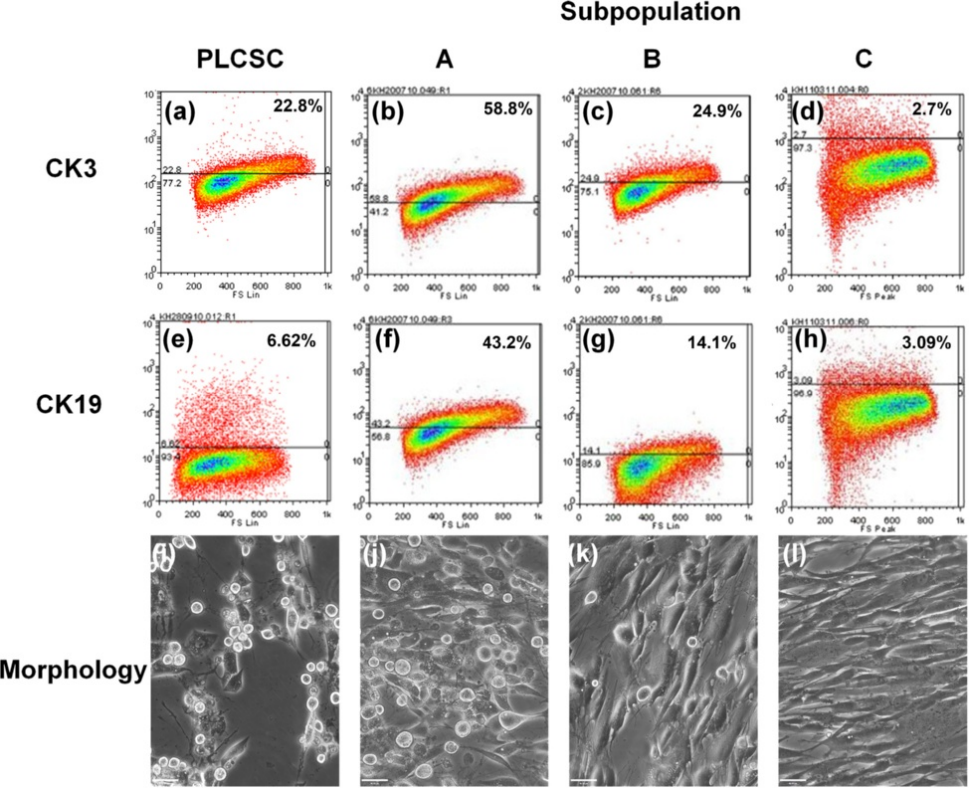
**Epithelial transdifferentiation of PLCSCs and subpopulations A, B, and C.** Epithelial differentiation of the entire PLCSC population **(a, e, i)** and subpopulations A **(b, f, j)**, B **(c, g, k)**, and C **(d, h, l)** was assessed. Analysis of percentage of cells expressing the differentiated epithelial marker CK3 (**a**, **b**, **c**, **d**) and the epithelial progenitor marker CK19 (**e**, **f**, **g**, **h**) was performed with flow cytometry on three separate donor cell populations. Images of cell morphology were taken after epithelial transdifferentiation (**i**, **j**, **k**, **l**). Magnification at 200×; scale bar, 46 μm.

PLCSCs were put through the procedure for epithelial differentiation (Figure 4) and subsequently analyzed by using flow cytometry for CK19 and CK3, markers of progenitor and differentiated corneal epithelium, respectively. PLCSCs expressed CK 19 in 7% and CK3 in 22.8% of cells. In an attempt to improve the yield/purity of epithelium produced by differentiation, PLCSC subpopulations were also analyzed for CK3 and CK19 (Figure 4). Subpopulation A showed 59% expression of CK3, the terminally differentiated marker, and 43% of the progenitor marker CK19, at day 14 of differentiation. Subpopulation B showed 25% expression of CK3 and 14% of CK19. Subpopulation C expressed CK3 and CK19 at low levels of 2.7% and 3.09% of cells, respectively. No coexpression of CK3 and CK19 was observed on differentiated cells (data not shown). In addition to marker expression, cell morphology changed, with an increase in adherent spherical cells, the prevalence of which correlated with increased numbers of cells expressing epithelial markers.

**Figure 5:**
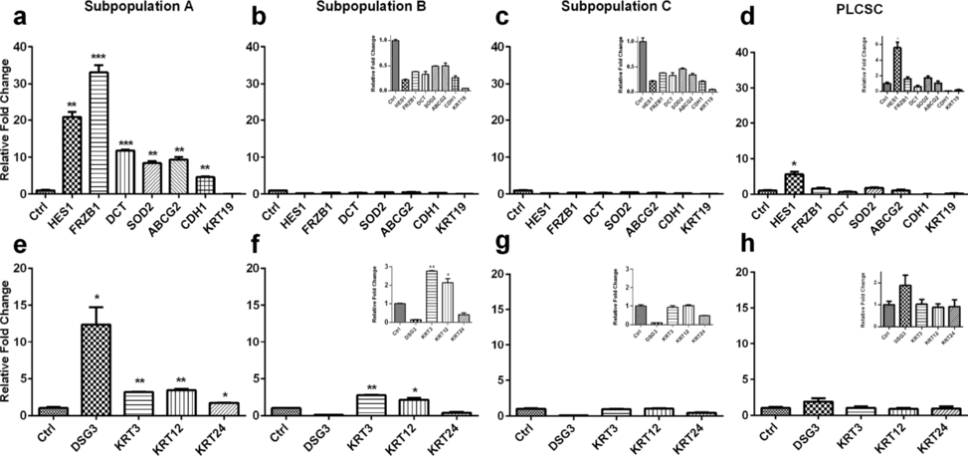
**Changes in gene expression after epithelial transdifferentiation of subpopulations A, B, C, and PLCSC populations.** Comparative qPCR was performed for epithelial progenitor genes (**a**, **b**, **c**, **d**, HES1, FRZ1, DCT, SOD2, ABCG2, CDH1, and KRT19) and terminally differentiated epithelial genes (**e**, **f**, **g**, **h** DSG3, KRT3, KRT12, KRT24). All fold changes are expressed relative to control gene 18S. Inset images show data on a reduced scale. Values are represented as mean ± SD. The experiment was repeated in triplicate, each with *n* = 3. Statistical significance is represented as *P ≤ 0.01, **P ≤ 0.001, ***P ≤ 0.0001.

Gene expression for epithelial progenitors (HES1, FRZB1, DCT, SOD2, ABCG2, CDH1, and KRT19) and terminally differentiated epithelial cells (DSG3, KRT3, KRT12, and KRT24) was assessed after epithelial differentiation of the three subpopulations (Figure 5). PLCSCs showed a significant 5.5-fold upregulation of the *HES1* gene, but not of any other progenitor or terminally differentiated genes. In contrast, subpopulation A showed increased expression for all corneal epithelial progenitor genes, with the exception of *KRT19. FRZB1* showed a 35-fold increase, and *HES1*, a 21-fold increase compared with control. Genes indicative of terminal differentiated corneal epithelium were also significantly upregulated compared with control, with the largest increase in *DSG3*, with a 12-fold expression. Subpopulation B showed no upregulation of the progenitor genes but did show a threefold upregulation of *KRT3* and *KRT12* for terminal differentiation markers. Subpopulation C showed no upregulation of either the progenitor or terminal-differentiation genes.

## Discussion

PLCSCs are a heterogeneous population of cells, containing several subpopulations, including the classic CD34^+^ keratocyte [66]. Previously, PLCSCs were characterized as CD34, ALDH, and keratocan positive, before culture (Branch *et al.*, 2012). The various subpopulations can be selectively isolated by using different culture media or by marker-specific isolation. Media selection of PLCSCs may have implications for the conventional understanding of keratocyte characteristics. Historically, retention and expansion of keratocyte numbers with a CD34^+^ phenotype *in vitro* has been difficult. Conventionally, the presence of serum or, more specifically, growth factors such as TGF-β1 [67,68] are reported to cause a fibroblastic or myofibroblastic phenotype, characterized by a fibroblast morphology and protein markers such as αSMA [68,69] and CD90 [70], along with the loss of keratocyte markers [6,71]. Some mitogen is essential for keratocyte growth, whether low serum or a serum substitute (such as defined growth factor combinations or bovine pituitary extract) is required for proliferation. Culture in low-mitogen or low-serum media has occasionally been reported to preserve keratocyte phenotype while promoting proliferation [18,67]; however, a new standard medium has yet to be established for this purpose. Notably, Funderburgh *et al.*[18,24] use low-serum or serum-free medium and described the multipotency of keratocytes and their precursors. Several other groups reported retention of keratocyte and stem cell phenotypes when culturing in DMEM/F12 [72-74] or DMEM/MCDB-201 [24,75] rather than DMEM, although factors such as low serum or addition of growth factors also cause variation.

Although keratocytes are routinely cultured in the classic DMEM-based medium, supplemented with 10% FBS and antibiotics [17,46,69,71,76,77] (referred to here as KM), our findings suggest that DMEM is a poor medium for the culture of keratocytes. Herein, we show that this medium causes a rapid loss of several markers, including CD105 and the keratocyte marker CD34. We recently characterized PLCSCs as MSC [15] (for which CD105 is a key marker) by using an M199-based medium (MM). However, when cultured in KM, PLCSCs do not conform to the MSC criteria, predominantly because of a loss of the CD105 marker. CD105 downregulation is associated with MSC differentiation [78,79], and the loss of CD105 observed in PLCSCs cultured in KM, therefore, indicates differentiation and a loss of stemness. This is supported by a decrease in multipotent differentiation potential when PLCSCs are cultured in KM. This loss of progenitor characteristics may explain why literature often describes keratocytes cultured in KM as activated, fibroblastic, and subsequently myofibroblastic in nature, but often does not discuss their progenitor properties [68,69,80].

The results of this study may also suggest that CD34 is a marker of corneal stromal progenitor cells similar to those described by Funderburgh *et al.*[18]. When cultured in MM, PLCSCs demonstrated greater retention of CD34 and CD105 markers at early passage, and conformed to the MSC criteria after subsequent passages. As PLCSCs cultured in MM have already been shown to conform to the ISCT’s established MSC criteria [15], this medium is useful for studying their multipotent associated characteristics.

By examining CSM expression, we have been able to investigate PLCSC heterogeneity further. We isolated and characterized three subpopulations of PLCSCs on the basis of CD34 and CD105 expression. Although no subpopulation conformed entirely to the ISCT guidelines for MSC characterization, the individual profiles of markers are not greatly dissimilar from MSC, indicating it is likely that they share a common, mesenchymal origin. Similar to PLCSCs cultured in MM, cells from subpopulation A, originally positive for CD34, give rise to cultures that lose observable CD34 expression. Our previous work indicated that this loss is due to slow-cycling CD34^+^ cells and the rapid proliferation of their progeny [15]. Although subpopulation A was originally CD34^+^ and subsequently contained a small number of CD45^+^ Cells. Despite this, subpopulation A did not produce hematopoietic colonies in specific HSC culture conditions (data not shown). Subpopulation A expressed stem cell markers, ABCG2 [81] and CD271 [82], not found in the other two subpopulations. CD271 is a known marker for osteogenic differentiation [82] and was expressed only by subpopulation A, the only group to demonstrate significant osteogenic differentiation. Subpopulations A, B, and C were also distinguished by the proportion of cells expressing CD13 and HLA ABC. Subpopulations B and C displayed similar marker profiles on further culture and reanalysis after sorting. The proportion of cells expressing CD105 decreased in subpopulation B, after isolation. However, when sorted for absence of CD105, subpopulation C consistently regained CD105 expression with further culture, reaching levels equivalent to subpopulation B. This contrasts with the irreversible loss of CD34 expression in subpopulation A. Subpopulation B possessed a greater capacity for differentiation than A with regard to adipogenesis; however, subpopulation A had the ability to differentiate into all three lineages, although adipogenesis was poor. Subpopulation C did not exhibit any trilineage potential.

Based on marker profiles and differentiation potential, our evidence suggests subpopulation A to be the most stemlike, and C, the most differentiated. However, isolated subpopulation C quickly reverts to a similar phenotype to subpopulation B, which suggests that the main difference lies in the presence or absence of CD34. Our results indicate that PLCSCs cultured in MM share similar characteristics with subpopulation A, whereas PLCSCs cultured in KM are phenotypically most similar to subpopulation C. With the appropriate optimization and development, culturing heterogeneous PLCSC populations in defined medium could be used to select a specific subpopulation of cells, with minimal contamination from other cell populations. This has considerable potential benefits, most significantly for clinical isolation and preparation.

The presence of so-called hematopoietic markers, such as CD34, on keratocytes [38,39,83] and other MSC-like cells [49,50,84-88] is not uncommon. CD34^+^ PLCSCs appear similar to the CD34^+^ multipotent progenitors found in fetal liver, which are able to produce mesenchymal lineages and biliary epithelium [49,51,89]. Both fetal liver and CD34^+^ PLCSCs generated CK19^+^ progenitors of biliary and corneal epithelium, respectively. MSC-like cells with these markers are often described as possessing potent stem cell properties, including the capacity to produce MSC [15,49,50,84-88] and epithelium [50,85]. MSC-epithelial transdifferentiation has been demonstrated in several studies [52-55]. CD34^+^ progenitors of keratinocytes in the skin are well documented [56-58]. We previously found CD34^+^ keratocytes that coexpressed the corneal epithelial marker CK3, indicating the potential for transdifferentiation from mesenchyme to epithelial lineages [59]. This demonstrates a strong association between CD34 and potential epithelial differentiation. This led to the resultant sorting and epithelial transdifferentiation of the three subpopulations with subsequent analysis at the gene and protein levels. Transdifferentiation of the different subpopulations revealed a varied capacity for the production of corneal epithelium. Subpopulation A, originally isolated as CD34^+^, demonstrated a rounded morphology, similar to that of corneal epithelium, after differentiation medium was applied. This subpopulation contained cells expressing CK19, an epithelial progenitor marker, and cells expressing CK3, a marker for terminally differentiated epithelial cells, indicating the presence of a mixed population of differentiated and undifferentiated corneal epithelium [13,90,91]. On further analysis, the cells of subpopulation A demonstrated an upregulation of epithelial progenitor genes (*HES1, FRZB1, DCT, SOD2, ABCG2, CDH1*, *and KRT19*) found in limbal crypt epithelium [14] and differentiated corneal epithelium genes (*desmoglein 3, KRT3, KRT12,* and *KRT24*) [13,14,90,91]. These markers were selected based on our recent body of evidence from transcriptomic characterization of corneal epithelial stems cells in the limbal epithelial crypts [14]. A comparatively lower percentage of cells from subpopulation B showed transdifferentiation into corneal epithelium, based on morphology and CK3 and CK19 expression, and, as a result, it was difficult to discern whether upregulation of corneal epithelial genes occurred, although CK3 and CK12 still demonstrated a significant increase in expression. Subpopulation C showed very little potential to undergo epithelial transdifferentiation, as assessed by gene, protein, and morphologic analysis.

## Conclusions

Our findings demonstrate that CD34^+^ PLCSCs have multipotent progenitor capacity, which includes differentiation into both mesenchymal and corneal epithelial phenotypes. This evidence challenges current perceptions of the role of the keratocyte, which may possess significant potential for corneal regeneration. However, current culture conditions are suboptimal for the expansion of these stemlike cells, and modest changes in formulation of culture medium can affect stemness. This development complements a growing body of literature confirming the presence and usefulness of stromal stem cells within the cornea [16-18,46]. Although novel with respect to the cornea, our findings are supported by similar observations in other parts of the body.

It is tempting to speculate that these corneal stromal stem cells contribute to the regeneration of the corneal epithelium *in vivo*. These cells are present in the limbal region, a proven site of epithelial stem cell activity [13,92], where the Bowman membrane, which normally functions as a barrier between epithelium and stroma, is absent [93,94]*.* Nevertheless, the CD34^+^ corneal stromal stem cells represent an exciting prospect for ocular tissue engineering and regenerative medicine. Specifically, they may provide an alternate cell source for the treatment of limbal stem cell deficiency.

## Abbreviations

ABCG: ATP-binding cassette subfamily G; ACAN: aggrecan; BMP: bone morphogenetic protein; CD: cluster of differentiation; CEC: corneal epithelial cell; CK: cytokeratin; COMP: cartilage oligomeric matrix protein; CSM: cell-surface marker; DCT: dopachrometautomerase; DMEM: dulbecco modified Eagle medium; DMSO: dimethylsulfoxide; DSG: desmoglein; FACS: fluorescence-activated cell sorting; FASN: fatty acid synthase; FBS: fetal bovine serum; FRZ: frizzled-related protein; HES: gairy and enhancer of split; HLA: human leukocyte antigen; HSC: hematopoietic stem cell; ISCT: international Society of Cellular Therapy; KM: keratocyte medium; KRT: keratin; LSC: limbal stem cell; MM: MSC medium; MSC: mesenchymal stem cell; OPG: osteoclastogenesis inhibitory factor; PLCSC: peripheral and limbal corneal stromal cell; PLIN: perilipin; PPARG: peroxisome proliferator-activated receptor-γ; qPCR: quantitative polymerase chain reaction; SOD: superoxide dismutase; SOX: Sry-related HMG box; TGF: transforming growth factor.

## Competing interests

The authors have no potential conflicts of interest.

## Authors’ contributions

KH participated in the cell culture, flow cytometry, differentiation, qPCR, and data analysis and assembly, and contributed to the concept and design. MJB participated in the flow cytometry, differentiation, cell culture, data analysis and assembly, and contributed to the concept and design and drafting of the manuscript. LES participated in the data analysis, microscopy, and assembly and drafting of the manuscript. MV participated in the qPCR and data analysis. PD participated in the flow cytometry, qPCR, and data analysis. ODM participated in the cell culture, flow cytometry, and differentiation. AH participated in the data analysis and assembly and contributed to the concept and design, drafted, and had final approval of the manuscript. HSD participated in the data analysis and assembly, contributed to the concept and design, and had final approval of the manuscript. All authors read and approved the final manuscript.
